# RLIM suppresses hepatocellular carcinogenesis by up-regulating p15 and p21

**DOI:** 10.18632/oncotarget.20904

**Published:** 2017-09-15

**Authors:** Yongsheng Huang, Meng Nie, Chuang Li, Yingjie Zhao, Jiahui Li, Lan Zhou, Lin Wang

**Affiliations:** ^1^ Department of Physiology, Peking Union Medical College, Chinese Academy of Medical Sciences, Institute of Basic Medical Sciences, Beijing 100005, China; ^2^ Department of Genetics, Albert Einstein College of Medicine, Bronx, NY 10461, U.S.A

**Keywords:** RLIM, hepatocellular carcinogenesis, p15, p21, MIZ1

## Abstract

Hepatocellular carcinogenesis results from dysregulation of oncogenes and tumor suppressors that influence cellular proliferation, differentiation and apoptosis. p15 and p21 are cyclin-dependent kinase inhibitors, which arrest cell proliferation and serve as critical tumor suppressors. Here we report that the E3 ubiquitin ligase RLIM expression is downregulated in hepatocellular carcinoma patients, and correlated with p15 and p21 expression in clinical progression. In addition, we showed that RLIM overexpression suppresses the cell growth and arrests cell cycle progression of hepatocellular carcinoma. Mechanistically, we found that RLIM directly binds to MIZ1, disrupting the interaction between c-MYC and MIZ1, and enhancing p15 and p21 transcription. Our results demonstrate that RLIM is an important suppressor in hepatocellular carcinogenesis.

## INTRODUCTION

Hepatocellular carcinoma (HCC), as the most prevalent type of primary liver cancers, is the third leading cause of cancer mortality worldwide [1, 2]. A number of HCC-related oncogenic genes and signaling pathways have been identified. However, the protein networks that regulate the expression and function of the HCC oncoproteins and tumor suppressors remain incompletely understood. Currently, surgical resection and chemotherapy are the two most commonly used strategies to treat HCC. However, because of insufficient hepatic reserve and poor response, both treatments fall short in providing a complete remission in the majority of the HCC patients. A better understanding of the regulatory network and discovery of new drug targets remains a top priority, in order to develop novel targeted therapies to better treat HCC.

*c-MYC*, one of the best-characterized oncogenes, is overexpressed in up to 70% of the HCC patients, and *c-MYC* amplification is often associated with poor prognosis. *c-MYC* inactivation was known to differentiate HCC cells into normal hepatocytes and biliary cells, while *c-MYC* reactivation restored the neoplastic features [3]. c-MYC is capable of both activating and repressing the transcription of target genes, many of which affect cancer development, through the interaction with different sets of transcriptional partners or co-factors [4]. To transactivate its downstream genes, c-MYC forms a heterodimer with Max, a ubiquitous co-factor, and binds to a consensus E-box site in the target promoter. For its transcription repressor activity, c-MYC, in complex with MIZ1 (MYC-interacting zinc-finger protein 1), recruits DNA methyltransferase DNMT3 and histone deacetylases to the promoter regions of targets genes, such as the cyclin-dependent kinase (CDK) inhibitor 2B (*CDKN2b*, encoding p15) and CDK inhibitor 1A (*CDKN1a*, encoding p21) [5, 6]. Small-molecule inhibitors that disrupt the c-MYC/Max heterodimerization or reduce *c-MYC* transcription have been developed in preclinical and clinical studies to treat advanced HCC [4, 7].

MIZ1 is a member of the poxvirus and zinc finger (POZ) domain protein family [8-10]. MIZ1 has 13 zinc fingers at its carboxyl terminus and an amino-terminal POZ domain, which is required for its transcriptional activity [9]. c-MYC interacts with a short helical domain in MIZ1, which is interspersed between zinc fingers 12 and 13 in the C-terminus of MIZ1 [9]. Similar to c-MYC, MIZ1 also plays a complex, dual role in regulating gene expression, i.e. functioning as either a transcriptional activator or repressor depending on its binding partners. For example, MIZ1, in a complex with co-factors such as nuclophosmin or p300, stimulates transcription of p21, p15 and Bcl-2, inhibitor of apoptosis [11, 12]. On the contrary, MIZ1 becomes a transcriptional repressor, when binding to c-MYC or Bcl-6, which replace nucleophosmin or p300, and represses the expression of p21 and p15 [5, 6]. In addition, MIZ1 can also interact with other oncoproteins such as Bcl-6 and Gfi-1, indirectly repressing the expression of the CDK inhibitors, and promotes cell proliferation or transformation [13, 14].

p15 and p21 interact with CDK complexes and block their kinase activities, thus prohibiting cell cycle progression and causing cell cycle arrest at G1 phase [15]. Aberrant expressions of p21 and p15 have been reported in a host of cancers, including HCC [16-20]. Since the c-MYC/MIZ1 complex represses the transcription of p15 and p21, higher levels and activity of the c-MYC/MIZ1 complex promotes carcinogenesis. Indeed, one recent study reported that c-MYC/MIZ1 promotes the proliferation of esophageal cancer cells through suppression of p21 [21]. Taken together, the critical transcriptional function of c-MYC and MIZ1 seem to heavily depend on the protein network, which these two factors reside in and interact with, and which in turn modulates their activities. At present, such protein networks and their mechanisms to fine-tune the transcriptional activities of c-MYC and MIZ1 are not well understood.

RLIM (RING finger LIM domain-binding protein) was originally identified as a LIM homeodomain (HD) binding protein, and inhibits the transcriptional activity of LIM-HD, thereby affecting embryogenesis and development [22], RLIM has also been implicated in X chromosome inactivation and the survival of female nurturing tissues in adult mice [23, 24]. In addition to functioning as a cofactor, RLIM also regulates the levels of multiple nuclear and cytosolic proteins, including CLIM/NLI/Ldb, Rex1, MDM2 and Stathmin, through its E3 ubiquitin ligase activity [25-28]. Several recent studies show that RLIM reduces cell proliferation, arrests cell cycle, and promotes cell migration in cancer cells. For example, RLIM could stabilize p53 and suppress breast cancer cell growth by targeting MDM2 for degradation [27]. We and others showed that RLIM positively regulates TGF-β signaling through Smurf2 and Smad7 [29, 30]. Because the TGF-β signaling pathway has a profound impact on carcinogenesis, RLIM is suspected to play a role in cancer development. At present, whether and how RLIM affects HCC is unknown.

In this study, we report that RLIM is lowly expressed in HCC tissues, compared to normal liver tissues. RLIM inhibits the proliferation and cell cycle progression of HCC cell lines. Moreover, RLIM directly binds to c-MYC and MIZ1, disrupting the c-MYC/MIZ1 complex, and increases the transcription of the downstream targets, p15 and p21.

## RESULTS

### The expression of RLIM is positively correlated with p15 and p21, and negatively correlated with the clinical progression of HCC

**Figure 1:**
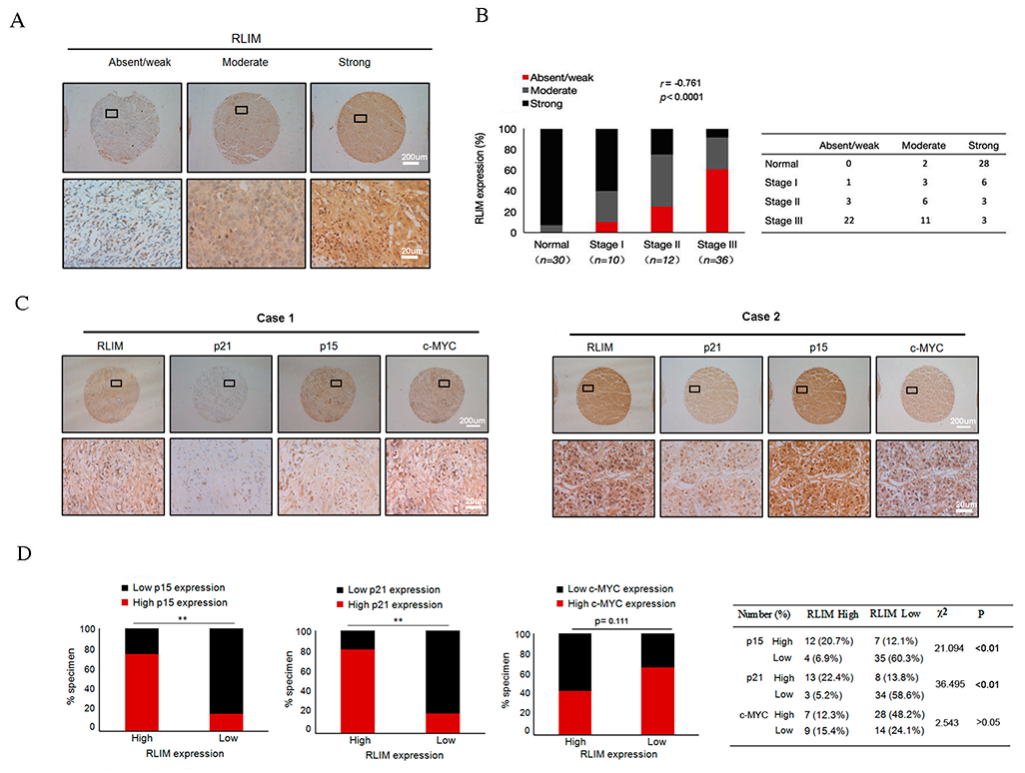
The expressions of RLIM negatively correlate with the clinical progression of HCC and positively correlate with the expressions of p15 and p21 **(A)** The representative IHC staining of RLIM in human normal liver and HCC tissues. The expressions of RLIM were classified as absent/weak, moderate and strong. Upper images are lower magnification, and lower images are enlarged insets. Scale bars, 200 μm; 20 μm (insets). **(B)** Left panel: analysis showing the percentages of RLIM expressions in normal liver tissues and each HCC clinical stage, with the r and p values of the Spearman rank correlation test indicated. Right panel: the number of different expressions of RLIM in human normal liver and HCC tissues. **(C)** The expressions of RLIM, p15, p21 and c-MYC were categorized as high and low. IHC analyses of the representative cases are shown. Upper images are lower magnification, and lower images are enlarged insets. Scale bars, 200 μm; 20 μm (insets). **(D)** Left panel: stacked bar graphs showing the percentages of specimens with either low or high expression of p15, p21 and c-MYC relative to RLIM level. **, *p<* 0.01. Right panel: the number and percentage of high and low p15, p21 and c-MYC expressions relative to RLIM expressions, including χ^2^ and p values from the Pearson’s chi-squared test.

To examine the correlation between RLIM expression and clinical progression of HCC, we performed IHC staining to detect the expression of RLIM in the liver tissues from 30 normal and 58 HCC patients with different clinical stages, including stage I (n =10), stage II (n =12), and stage III (n=36). We found a strong negative correlation (r =-0.761, p < 0.0001) of RLIM levels with HCC progression (Figure 1A, 1B). We have also similarly detected the expressions of p15, p21 and c-MYC in HCC tissue microarray. Consistent with several previous reports [31-33], the levels of p15 and p21 exhibited a strong negative correlation (r = -0.586, p < 0.0001 and r =-0.473, p < 0.0001, respectively) with the clinical progression of HCC, while the expressions of c-MYC showed a less significant and positive correlation (r = 0.309, p = 0.003) (Supplementary Figure 1A-1F). Next, we examined any correlation between the expression of p15, p21 and c-MYC and RLIM levels in normal and HCC tissues (Figure 1C, 1D). We found that the levels of RLIM exhibited a strong correlation with the expressions of p15 and p21, but not with c-MYC (p < 0.01 versus p > 0.05) (Figure 1D). The c-MYC immunostaining on biopsy samples confirmed that c-MYC was markedly overexpressed in HCC, compared with normal liver tissues (Supplementary Figure 2). Taken together, these data suggest that RLIM might be an important repressor in the hepatocellular carcinogenesis and might be functionally linked to the expression of p15 and p21.

### RLIM enhances p15 and p21 expression through c-MYC/MIZ1

**Figure 2:**
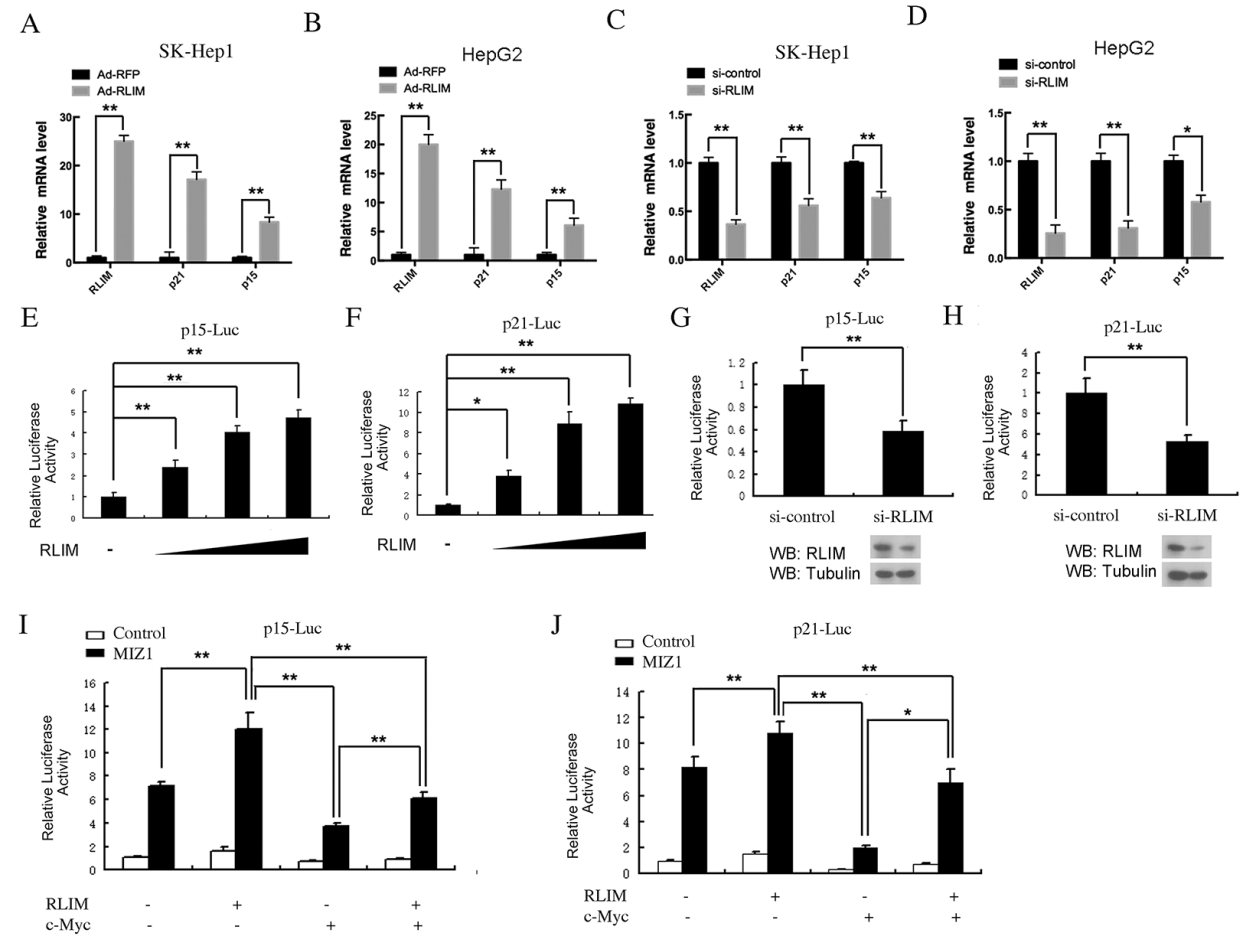
RLIM enhances p15 and p21 expression through MIZ1 *in vivo* **(A-B)** Overexpression of RLIM increases p15 and p21 gene expression in HCC cells. SK-Hep1 (A) and HepG2 (B) cells were infected with recombinant RLIM or RFP adenovirus for 48 h and then analyzed for real time-qPCR. **(C-D)** Silencing of RLIM decreases p15 and p21 gene expression in SK-Hep1 (C) and HepG2 (D) cells. **(E-F)** RLIM increases the expression of p15 and p21 luciferase reporter in a dose-dependent manner. HEK 293T cells were transfected with 0.2 μg of p15-Luc (E) or p21-Luc (F), and varying amounts of a RLIM construct (0, 0.1, 0.2, 0.3 μg). 48 h after transfection, the cells were examined for luciferase activity. **(G-H)** Silencing of RLIM reduces the expression of p15-Luc (G) and p21-Luc (H) in HEK 293T cells. **(I-J)** RLIM affects the expression of p15-Luc (I) and p21-Luc (J) through c-MYC and MIZ1. HEK 293T cells were co-transfected with p15-Luc and p21-Luc, with RLIM, c-MYC and MIZ1 constructs as indicated, and 48 h after transfection, the cells were examined for luciferase measurement. All the experiments were performed with co-transfection of Renilla-luciferase (20 ng) as an internal control. The data were derived from three independent experiments and expressed as mean + SEM, **, *p<* 0.01; *, *p<* 0.05.

Next, we examined the correlation between RLIM and p15/p21 expression in the HCC cell lines by quantitative RT-PCR. We used recombinant adenovirus to overexpress RLIM or RFP in SK-Hep1 and HepG2 cells, two classic HCC cell lines that express high levels of c-MYC. We observed a marked induction of p15 and p21 mRNAs after overexpression of RLIM in both cell lines (Figure 2A, 2B). In a reverse experiment, we observed a significant decrease of p15 and p21 mRNAs after silencing of RLIM in these cells (Figure 2C, 2D). Furthermore, we confirmed the effect of RLIM on the transcriptional activation of p15 and p21 using the luciferase reporters, p15-Luc and p21-Luc, in HEK 293T cells (Figure 2E, 2F). HEK 293T kidney cells were chosen for the ectopic expression in our study, primarily because they are more amendable to transfection and provides a non-hepatic expression system to examine the role of RLIM in the transcriptional activation of p21 and p15. Overexpression of RLIM enhanced the reporter activities of p15 and p21 in a dose-dependent manner (Figure 2E, 2F). Consistently, silencing of endogenous RLIM by small interfering RNAs (siRNAs) in HEK 293T cells led to lower reporter activities (Figure 2G, 2H). Because the efficiency of RNA silencing with RLIM siRNAs was not high (estimated to around 60% and 70%, respectively, based on the RLIM protein level), the effect on the luciferase activity was moderate, compared with the effect of overexpression.

Interestingly, the transcription of p15-Luc and p21-Luc were greatly enhanced, when MIZ1 was co-expressed in HEK 293T cells (Figure 2I, 2J). The co-expression with RLIM further enhanced the transcriptional activities of p15-Luc and p21-Luc in HEK 293T cells, suggesting that RLIM might have a synergistic effect with MIZ1. Notably, the sole co-expression of c-MYC produced the opposite effect, i.e. inhibiting the expression of p15-Luc and p21-Luc instead (Figure 2I, 2J). Consistent with the previous reports, co-expression of c-MYC with MIZ1 reduced the expression of p15-Luc and p21-Luc, compared with the sole co-expression of MIZ1. Furthermore, the additional co-expression of RLIM reversed the c-MYC-mediated inhibition on the transcriptional activation of MIZ1 on p15-Luc and p21-Luc (Figure 2I, 2J). Taken together, these results strongly suggest that RLIM interacts with both MIZ1 and c-MYC to regulate the transcription of p15 and p21. These results also highlight the dual nature of MIZ1 in regulating the transcription of these CDK inhibitors.

### RLIM interacts with MIZ1 and c-MYC

**Figure 3:**
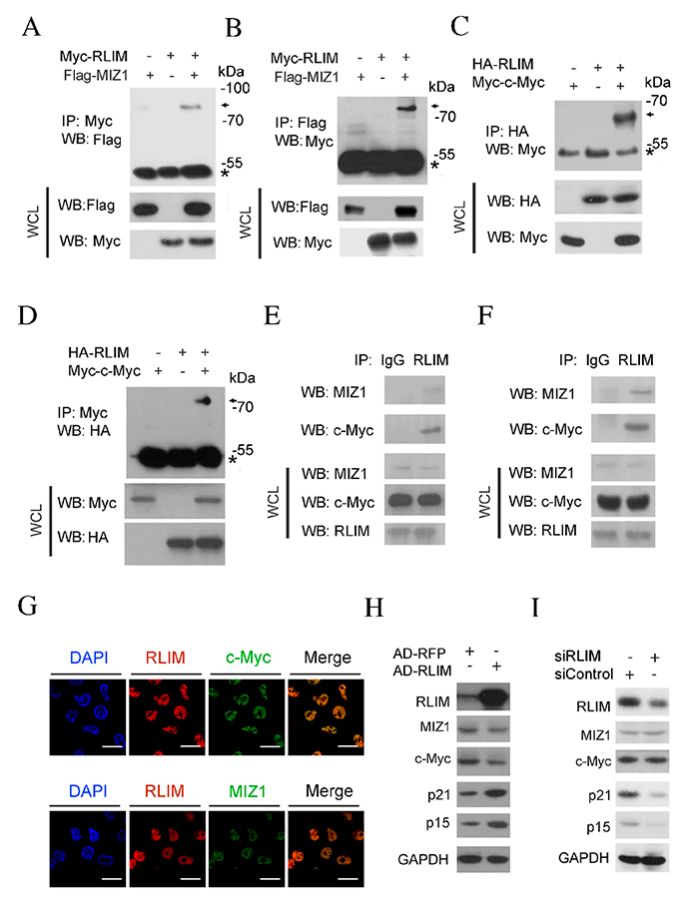
RLIM interacts with MIZ1 and c-MYC **(A-B)** HEK 293T cells were co-transfected with Myc-RLIM (MW: 72KDa) and Flag-MIZ1 (MW: 80KDa). 48 h post-transfection, cells were lysed and subjected to immunoprecipitation (IP) with Myc (A) or Flag (B) antibodies, respectively. The immunoprecipitates and whole cell lysate (WCL) were analyzed by immunoblotting. * indicates the position of IgG heavy chain. **(C-D)** HEK 293T cells were co-transfected with HA-RLIM (MW: 72KDa) and Myc-c-MYC (MW: 65KDa). 48 h post-transfection, cells were harvested and subjected to IP with HA (C) or Myc (D) antibodies, respectively. **(E-F)** IP of endogenous RLIM protein from SK-Hep1 (E) and HepG2 (F) cells. The associated c-MYC and MIZ1 proteins were analyzed by immunoblotting. **(G)** SK-Hep1 cells were fixed and immunostained with the fluorescently labeled antibodies against RLIM (red) and c-MYC or MIZ1 (green). The nuclei were counterstained with DAPI (blue). Scale bars: 50 μm. **(H)** SK-Hep1 cells were infected with RFP or RLIM recombinant adenovirus. Endogenous proteins were analyzed by immunoblotting with indicated antibodies. **(I)** SK-Hep1 cells were transfected with scramble siRNAs or siRNAs against RLIM. Endogenous proteins were analyzed by immunoblotting with indicated antibodies.

Co-immunoprecipitation (Co-IP) was used to confirm the interaction between RLIM and c-MYC and MIZ1. Myc or Flag tagged chimeric constructs, Myc-RLIM and Flag-MIZ1, were expressed into HEK 293T cells by transient transfection. 48 hours after transfection, cells were lysed and immunoprecipitation was carried out. As shown in Figure 3A, Myc antibody successfully co-precipitated Flag-MIZ1. In a reciprocal experiment, we showed that antibody against Flag was able to pull down Myc-RLIM (Figure 3B). These results demonstrate that the ectopically expressed MIZ1 and RLIM directly interacted with each other. Similarly, we found that the Myc or HA tagged chimeric constructs of c-MYC and RLIM interacted with each other, when ectopically expressed in HEK 293T cells (Figure 3C, 3D). Then we confirmed the interaction between the endogenous RLIM and c-MYC or MIZ1 in HCC cells. The endogenous RLIM was immunoprecipitated from the SK-Hep1 and HepG2 cell lysates. The endogenous c-MYC and MIZ1 was found to co-precipitate with RLIM (Figure 3E, 3F). Furthermore, immunofluorescence was performed to examine the intracellular location of RLIM and c-MYC or MIZ1 in SK-Hep1 cells. Figure 3G shows that the majority of RLIM co-localized with c-MYC and MIZ1 in the nucleus of SK-Hep1 cells.

From the above data, we suspected that RLIM might enhance the transcription of p15 and p21 in a c-MYC/MIZ1 dependent manner. To test this possibility, we examined whether overexpression of RLIM affected the p15 and p21 protein level. As shown in Figure 3H, the protein levels of p15 and p21 were greatly elevated, when RLIM was overexpressed in SK-Hep1 cells. On the contrary, the protein levels of p15 and p21 decreased, when RLIM was silenced (Figure 3I). Interestingly, the overexpression or silencing of RLIM levels did not significantly affect the protein levels of c-MYC or MIZ1 (Figure 3H, 3I). Our finding that RLIM did not affect c-MYC levels was consistent with a previous report [34].

### RLIM disrupts the c-MYC/MIZ1 interaction

**Figure 4:**
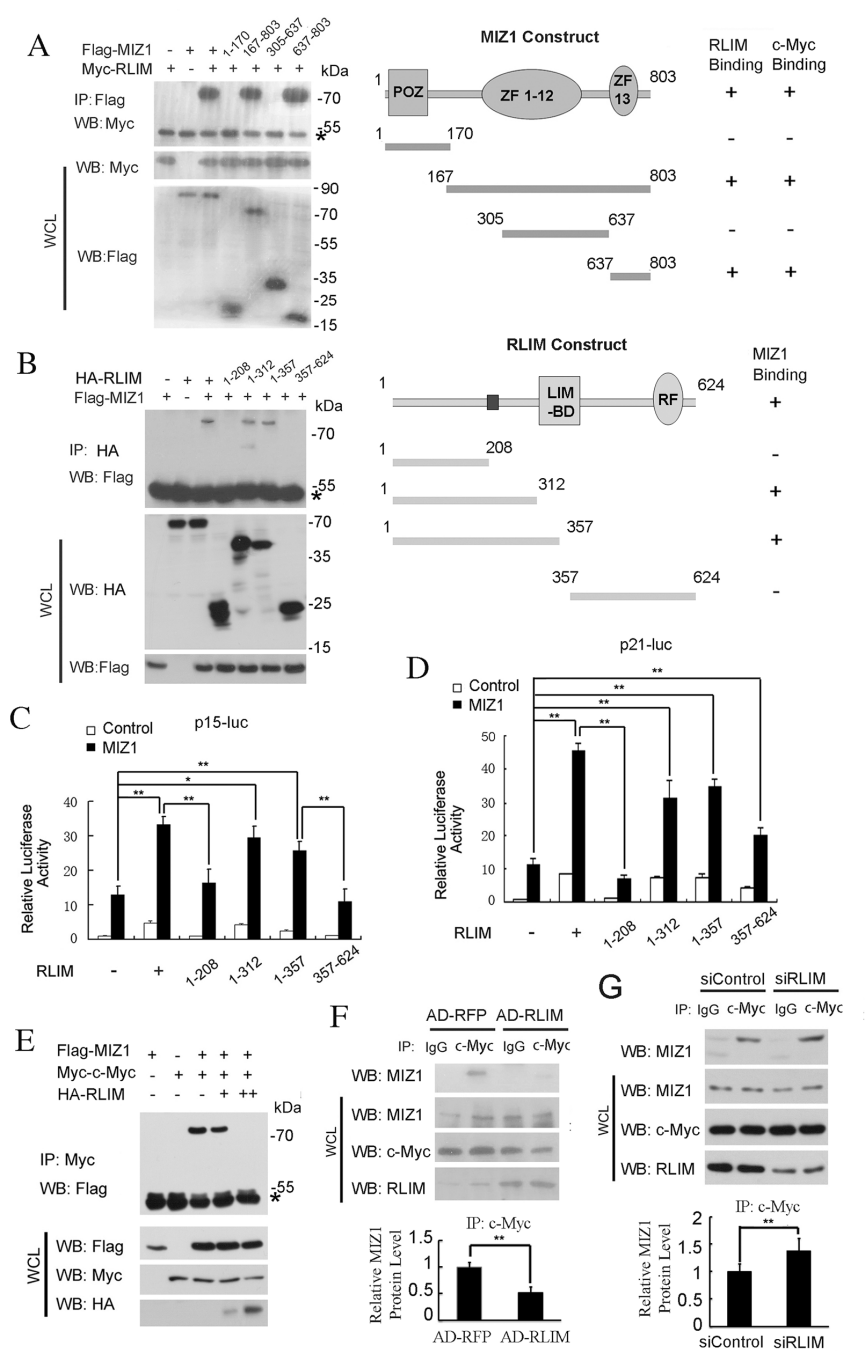
RLIM disrupts the c-MYC/MIZ1 interaction **(A)** Left panel: Mapping the MIZ1 domains to interact with RLIM. HEK 293T cells were transfected with indicated constructs and their interaction was examined by immunoprecipitation (IP) and immunoblotting with indicated antibodies. Right panel: a schematic diagram of MIZ1 truncation constructs, which were taken from reference [9], and their interactions with RLIM. POZ and zinc fingers (ZF) 1-12 and 13 were indicated. **(B)** Left panel: Mapping the RLIM domains to interact with MIZ1. Right panel: a schematic diagram of RLIM truncation constructs and their interactions with MIZ1. LIM-binding domain (LIM-BD) and ring finger (RF) were indicated. **(C-D)** The differential effects of RLIM truncation constructs on the expression of p15-Luc (C) and p21-Luc (D). **(E)** The interaction between the exogenously expressed c-MYC and MIZ1 was reduced by the co-expression of HA-RLIM. HEK 293T cells were co-transfected with Flag-MIZ1 and Myc-c-MYC, with or without HA-RLIM (+: 1 μg; ++: 2 μg). **(F-G)** IP of the endogenous c-MYC protein in SK-Hep1 cells with RLIM overexpressed by recombinant adenovirus infection (F) or RLIM silenced with siRNAs (G). The co-precipitated MIZ1 was analyzed by immunoblotting and the quantitation was shown in the bottom panels. The data were derived from three independent experiments and expressed as mean + SEM, **, *p*< 0.01; *, *p*< 0.05.

We next mapped out the domains in MIZ1 to interact with RLIM using a series of truncation fragments of MIZ1, and found that a C terminal region (637–803aa) of MIZ1 is essential and sufficient to bind to RLIM (Figure 4A). The same region has previously been shown to mediate the interaction between MIZ1 and c-MYC [9]. Similarly, we used a series of truncation fragments of RLIM and mapped out a middle region in RLIM (208-312aa) that was responsible for the interaction with MIZ1 (Figure 4B). These data, together with the previous report [9], suggest that RLIM and c-MYC may bind to the same C terminal region (637–803aa) region of MIZ1 in a competitive manner. Thus, it is plausible that RLIM may enhance the transcription of p15 and p21 by disrupting the c-MYC/MIZ1 interaction.

Indeed, the truncation constructs encoding the middle region of RLIM protein (208-312aa) also increased the expression of p15-Luc and p21-Luc, whereas other constructs lacking this region failed to stimulate (Figure 4C, 4D). Furthermore, the interaction between Flag-MIZ1 and Myc-c-MYC was reduced by the co-expression of HA-RLIM in a dose-dependent manner in HEK 293T cells (Figure 4E). Consistently, we also found that the co-precipitation of the endogenous MIZ1 with c-MYC (i.e. the interaction between the endogenous c-MYC and MIZ1) was reduced in SK-Hep1 cells, when RLIM is overexpressed (Figure 4F). On the other hand, the interaction between the endogenous MIZ and c-MYC was increased in SK-Hep1 cells, when RLIM was silenced (Figure 4G). Because the efficiency of RNA silencing of RLIM was not high enough, the increase of the co-precipitated MIZ1 with c-MYC was moderate.

### RLIM suppresses HCC cell proliferation and cell cycle

**Figure 5:**
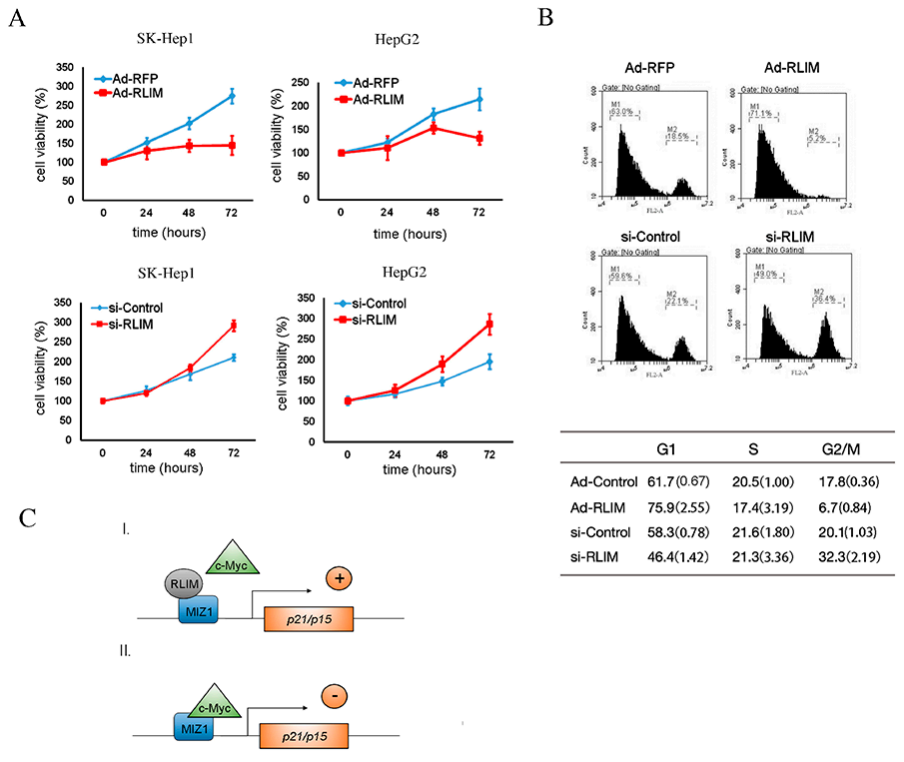
RLIM suppresses HCC cell proliferation and cell cycle progression **(A)** SK-Hep1 and HepG2 cells were infected with RLIM or RFP recombinant adenovirus. The cell growth were monitored by MTS assay at the indicated time points and expressed as mean ± SEM from triplicate experiments. **(B)** SK-Hep1 and HepG2 cells were infected with RLIM or RFP recombinant adenovirus for 18 h and then subjected to cell synchronization, followed by flow cytometry analysis. The data in the table were derived from three independent experiments, and represent the mean (SEM in bracket) from triplicate experiments. **(C)** A working model illustrating the mode of action for RLIM in the transcriptional regulation of p15 and p21, through interaction with the c-MYC/MIZ1 complex.

Given that RLIM interfered with the interaction between c-MYC and MIZ1 and thus enhanced the transcription of p15 and p21, we wondered whether the levels of RLIM would affect the cell proliferation and cell cycle progression. To investigate this, we stably overexpressed RLIM in SK-Hep1 and HepG2 cells with recombinant adenovirus and measured cell proliferation. As shown in Figure 5A, RLIM overexpression suppressed the proliferation of SK-Hep1 and HepG2 cells, while silencing of RLIM promoted the proliferative ability of SK-Hep1 and HepG2 cells (Figure 5A). Consistently, overexpression of RLIM increased the cell number in G1 phase, but decreased the cell number in G2/M phase in both SK-Hep1 and HepG2 cells, while silencing of RLIM produced the opposite effect (Figure 5B). These data together demonstrated that higher levels of RLIM inhibited HCC cell proliferation and cell cycle progression.

## DISCUSSION

In this study, we, for the first time, demonstrate that RLIM functions as a tumor suppressor in HCC. We found that RLIM expression was low in HCC tissues and exhibited a strong negative correlation with the clinical progression of malignancy. The levels of p15 and p21 also showed a strong negative correlation with the HCC clinical progression, while the correlation with c-MYC expressions was less strong. This is interesting, because HCC is a heterologous disease and can be driven by multiple dysregulated signaling pathways. Indeed, high c-MYC expression is only seen in up to 70% of the HCC patients, which may explain the less strong association of c-MYC with HCC clinical progression. The strong association of RLIM with HCC clinical progression seems to suggest that RLIM may function as a suppressor for multiple signaling pathways, in addition to c-MYC. For example, many HCC patients carry mutations in β-catenin, which often lead to aberrant WNT signaling [35]. The levels of c-MYC, a downstream target of β-catenin, do not always reflect the changes in β-catenin [36, 37]. At present, there is no correlation study between RLIM and β-catenin, although it is plausible that RLIM may also function as a suppressor in the β-catenin/WNT pathway.

Our IHC study further showed that the levels of RLIM positively correlated with the expression of p15 and p21, but not with c-MYC. We then confirmed that RLIM repressed the transcription of p15 and p21 in two HCC cell lines. Using luciferase reporter constructs, we further showed that the co-expression of RLIM enhanced the stimulating effect on the transcription of p15 and p21 by MIZ1, while reversing the inhibitory effect on the transcription of p15 and p21 by c-MYC. This is very interesting, because RLIM is known to promote or inhibit the activity of transcription factors in a context-dependent manner. For example, RLIM inhibits the transcriptional activity of LIM-HDs, while enhancing transcriptional activation of endogenous ERα target genes [22, 38]. Thus, these results suggest that the regulation of RLIM on p15 and p21 transcription is also dependent on the presence of different set of cofactors.

These data also strongly indicated that RLIM physically interacts with MIZ1 and c-MYC. We went on to demonstrate that RLIM directly associates with MIZ1 and c-MYC and RLIM disrupts the MIZ1/c-MYC complex through competitive binding with c-MYC at the same C-terminal region of the binding of MIZ1. The region in RLIM (208-312aa) involved in RLIM-MIZ1 interaction is interesting. RLIM is known to shuttle between the nucleus and cytoplasm, and contains a 209–230aa nuclear localization sequence (NLS) [39]. We generally assume that the residues involved in RLIM-MIZ1 interaction do not overlap with the NLS, which contains stretches of basic residues and is specifically responsible for nuclear import. We speculate that the RLIM region (230-312aa) C terminal to the NLS is responsible for the RLIM-MIZ1 interaction.

A search using Conserved Domains of NCBI indicated that, in addition to the RING finger in the C terminus, two regions of RLIM bear significance to known protein motifs and overlap with the region 231-312aa. First, the RLIM region (148-249aa) is homologous to the C terminal region of a polarity protein Dishevelled, which has been previously demonstrated to interact with MIZ1 through its PDZ domain [40]. Secondly, the region (159-348aa) is homologous to C-terminal region of herpes virus ICP4 (infected-cell polypeptide 4)-like protein. ICP4 is required for efficient transcription of early and late viral genes, and is known to interact with viral and transcriptional proteins through the concerted action of its C-terminal and N-terminal regions. Whether the residues in these two RLIM regions play any role in the RLIM-MIZ1 interaction requires further investigation.

Only one recent study reported the link of RLIM with c-MYC [34]. RLIM associates with c-MYC in HEK 293T and human lung cancer H1299 cells, independently of its E3 ligase activity [34]. RLIM promotes the polyubiquitination of c-MYC, while not affecting its stability. RLIM inhibits the transcriptional activity of c-MYC and suppresses cell proliferation. Our result that RLIM interacted with c-MYC and reversed the c-MYC-mediated inhibitory effect on p15 and p21 transcription is entirely consistent with this recent report. Notably, our study also illustrated the additional regulatory mechanism of RLIM on MIZ1, the novel interactions within the RLIM-MIZ1-c-MYC tripartite complex, and the concerted action of such a complex on transcription of p15 and p21. Indeed, there was no report on the relationship between RLIM and MIZ1, prior to our study.

Mechanistically, c-MYC forms a heterodimer with Max to transactivate its downstream genes. It remains to be determined whether RLIM also binds to Max and affects the c-MYC/Max complex. The c-MYC-activated downstream genes in HCC include human telomerase reverse transcriptase, and vascular-endothelial growth factor-A (VEGFA), which is regulated by HIF-1α in corporation with c-MYC [41, 42]. Whether RLIM affects the expression of these downstream target genes also remains to be determined. The binding of c-MYC to MIZ1 replaces nucleophosmin or p300 [11, 12], and after binding to MIZ1, c-Myc recruits DNA methyltransferase DNMT3 to p21 promoter to silence p21 transcription [43]. Clearly, future studies are also required to characterize the impact of RLIM on these mechanistic steps of transcriptional regulation by the MIZ1/c-MYC complex. Interestingly, in addition to the presumed nuclear location, MIZ1 was also shown to interact with Dapper1 and Dishevelled, cytosolic effectors of Wnt signaling pathway, and promote colon cancer proliferation [40].

In summary, this study reported that tumor suppressor role of RLIM in HCC, and presented evidence on the underlying mechanisms, whereby RLIM interacts with MIZ1 and c-MYC, regulating the transcription of p15 and p21 and influencing the proliferation and cell cycle progression in hepatic carcinogenesis. These findings provide a layer of previously unreported regulation for HCC development and likely have general significance for other cancers as well. Our study also suggest that RLIM is a potential drug target for future targeted therapies against HCC and especially those characterized by higher c-MYC expression.

## MATERIALS AND METHODS

### Cell culture and transfection

SK-Hep1, HepG2 and HEK 293T cells were from the Cell Resource Center of Chinese Academy of Medical Sciences, and were maintained in Dulbecco’s modified Eagle’s medium (DMEM) containing 10% fetal bovine serum (Gibco, California, USA) and 100 U/ml penicillin-streptomycin (Invitrogen, Carlsbad, California, USA) at 37 °C with 5% CO_2_. Cells were transfected with Lipofectamine 2000 (Invitrogen) or VigoFect (Vigorous, Beijing, China), following the manufacturer’s instructions.

### Antibodies and reagents

Antibody against RLIM (16121-1-AP) was purchased from Proteintech (Chicago, Illinois, USA). Antibodies against p15 (#4822), p21 (#2947) and c-MYC (#5605) were purchased from Cell Signaling Technology (Boston, Massachusetts, USA). Antibodies against MIZ1 (sc-136985) and c-MYC (sc-40) were purchased from Santa Cruz Biotechnology (California USA). Antibodies against HA (M180-3), myc (M047-3), Flag (M185-3L) and GAPDH (M171-3) were purchased from MBL (Nagoya, Japan). All other chemicals were purchased from Sigma-Aldrich, unless stated otherwise.

### Plasmids and siRNA

The RLIM cDNA was amplified from Marathon fetal liver cDNA library (Takara, Mountain View, California, USA), and subcloned into pCMV-HA/Myc vectors. The pUHD-MIZ1 was kindly provided by Dr. Frank Hanel (Hans Knoell Institute, Germany), and was subcloned into pCDNA3.1+ with an N-terminal Flag tag. All truncation mutants were generated using the KOD-Plus Mutagenesis Kit of Toyobo (Osaka, Japan). The truncation constructs of RLIM and MIZ1 were also subcloned into pCMV-HA/Myc and pCDNA3.1-Flag vector, respectively. Two siRNA oligonucleotides against RLIM were purchased from GenePharma (Shanghai, China) with sequences as follows, siRNA1: 5’-GUUCCAGUUCCAGUCCUAG-3’ and siRNA2: 5’-CACUUGCUCCUCCAAAAUC-3’.

### Adenoviral expression

Recombinant adenovirus were prepared with the Adenoviral Vector System as previously described (Stratagene, California, USA). The coding regions of RLIM and red fluorescent protein (RFP) were first subcloned into pShuttle–CMV vector, and subsequently linearized by digestion with *Pme*I. The linearized plasmids were then co-transformed into BJ5183 cells with pAdEasy-1, an adenoviral backbone plasmid. Recombinants were selected and amplified in HEK 293A cells. High-titer viral stocks were purified by CsCl banding; final yields were generally at 10^11^ to 10^12^ plaque-forming units. Procedures for CsCl banding and viral plaquing have been described previously [44].

### Immunohistochemistry

The tissue microarrays used for correlation analysis of RLIM expressions were purchased from Alenabio Inc (Xi’an, China), and consist of stage I-III HCC and normal liver tissues (n=88). Alenabio Inc collected these surgically resected tissues, under the highest ethical standards, and with the donors being completely informed and their consent requested. The tissue microarray sections were deparaffinized and rehydrated, then the endogenous peroxidase was inactivated with a 3% hydrogen peroxide/methanol solution, followed by washing with a phosphate buffer solution (PBS). After incubation for 15 min in a boiling water bath for antigen retrieval, the sections were incubated with the primary antibodies against RLIM, p15, p21 and c-MYC at 4°C overnight, respectively, followed by rinse with PBS and further incubation with the biotinylated secondary antibody (Jackson Immuno Research Laboratories Inc., Pennsylvania, USA) for 1 h at room temperature. The proteins of interest were visualized with a liquid DAB substrate-chromogen system (ZSGB-BIO, Beijing, China). The images of immunohistochemical (IHC) staining were captured by a Leica microscope (Solms, Germany).

The expression levels of RLIM, p15, p21 and c-MYC were determined by the mean of the percentage of the positive cells from 5 randomly selected fields in each spot of the tissue microarray. The cutoff values for absent/weak, moderate and strong expressions were defined as 0-30%, 30-60% and 60-100%, respectively. To examine the correlation of RLIM, p15, p21 and c-MYC expressions, their expressions were artificially categorized as low (0%–50%) and high (50%–100%). All the stainings were examined by three observers (M.N., C.L. and Y.H.). The significance of correlation between RLIM level and HCC clinical stages was evaluated using the Spearman rank correlation test. The significance of correlation between RLIM and p21 or p15 was determined by the Pearson’s Chi-squared (χ^2^) test.

### Immunoprecipitation and immunoblotting

Briefly, cells were lysed with a lysis buffer (20 mM Tris-HCl pH 7.4, 2 mM EDTA, 25 mM NaF and 1% Triton X-100), containing protease inhibitors (Roche, Basel, Switzerland). Then the lysates were centrifuged at 4°C for 5 min at 12,000 g, and the supernatant was incubated with specific antibody and protein A/G beads overnight at 4°C. Next day, the precipitants were washed 3 times with washing buffer and eluted with sample buffer for 5 min at 95°C. Immunoprecipitated proteins were separated by SDS-polyacrylamide gel electrophoresis (SDS-PAGE) and transferred to nitrocellulose membranes (Pall, Ann Arbor, Michigan, USA). The membrane was incubated with a primary antibody overnight at 4°C, followed by incubation with secondary antibody for 1 h at room temperature. Finally, the proteins of interest were detected using ECL chemiluminescence (Santa Cruz).

### Immunofluorescence

Cells were fixed for 10 min in PBS containing 4% paraformaldehyde and then permeabilized with 0.2% Triton X-100. Next, the cells were blocked in PBS containing 5% bovine serum albumin for 30 min at room temperature. Afterwards, the cells were incubated with primary antibodies at 4^°^C overnight. The cells were washed 3 times in PBS, then incubated with fluorescein isothiocyanate (FITC)-conjugated or tetramethylrhodamine β-isothiocyanate (TRITC)-labeled secondary antibodies for 1 h at room temperature. The nuclei were counterstained with DAPI. Immunofluorescent images were captured by a confocal microscope FV1200 (Olympus, Tokyo, Japan).

### Real-time quantitative PCR (qPCR)

Total RNAs were extracted in Trizol (Life Technologies, Massachusetts, USA). 2 μg of total RNA was reverse transcribed to generate cDNA, using cDNA synthesis kit (TransGene, Beijing, China). Real-time qPCR analysis was performed in triplicates, with GAPDH as internal controls, using SYBR Green qPCR SuperMix and the StepOnePlus (Applied Bio-systems, California, USA) Real-Time Detection System and following the manufacturer’s instructions. The PCR primers were designed using Primer 3, and their specificity was verified using BLAST (NCBI, Maryland, USA). Primers used in this study are as follows: RLIM-f, TGAGAGATAACAATTTGCTAGGC and RLIM-r, GTGGGCCTTCTTTAATTTGC; p21-f, TGTCCGCGAGGATGCGTGTTC and p21-r, GCAGCCCGCCATTAGCGCAT; p15-f, AGATCCCAACGCCCTGAAC and p15-r, CCCATCATCATGACCTGGATT; GAPDH-f, GAGTCAACGGATTTGGTCGT and GAPDH-r, GACAAGCTTCCCGTTCTCAG.

### Transcription reporter assay

The luciferase reporter constructs under the human p15 promoter (-2.5 kb ∼ +0.16 kb) and p21 promoter (-2.4 kb ∼ +0.01 kb) were kindly provided by Prof. Ye-Guang Chen (Tsinghua University, Beijing, China). HEK 293T cells were seeded at a density of 8×10^4^ cells per well in 24 well plates and transfected with various amounts of plasmids as indicated in the figures. Transient transfection was performed with VigoFect (Vigorous, Beijing, China). 48 h after transfection, the cells were harvested and luciferase activities were measured by a luminometer (Berthold Technologies, Stuttgart, Germany). The internal control Renilla activity was used to normalize the luciferase activity. Each assay was performed in triplicate and the data represent the mean ± s.e. of three independent experiments.

### Cell proliferation assay

The cells were plated in 6 replicates at a density of 3×10^3^ cells per well in a 96 well plate and infected by adenoviral stocks. Cell proliferation was monitored by the CellTiter 96 AQueous One solution Cell Proliferation Assay (MTS) as instructed (Promega, Wisconsin, USA). The number of live cells was determined from optical absorbance at 490 nm in a microplate reader (Biotek, Vermont, USA), and the measurement was conducted every 24 h for continued 3 days.

### Cell synchronization and FACS analysis

To monitor cell cycle phases, cells were treated with 2.5 mM thymidine for 20 h and cultured with fresh medium for next 10 h. Then, cells were treated with 50 nM Nocodazole for another 10 h and fixed in 4% formaldehyde for 10 min at 37°C. Then, cells were stained with propidium iodide (50 μg/mL) containing RNAase (100 μg/ml) for 10 min at 37°C. Finally, the cell cycle was analyzed using a flow cytometer (BD, New Jersey, USA).

### Statistical analysis

Experiments were repeated at least three times, and all data were presented as mean + or ± standard error of the mean (SEM). Significant differences were determined by using the two-tailed student’s t-test, where a value of *, *p* ≤ 0.05 and **, *p* ≤ 0.01 was considered statistically significant. The significance of correlation between the levels of RLIM, p15, p21 and c-MYC and HCC clinical stages was evaluated using the Spearman rank correlation test [45]. The significance of correlation between RLIM and p15, p21 or c-MYC was determined by the Pearson’s chi-squared (χ^2^) test [46].

## SUPPLEMENTARY MATERIALS FIGURES


