# Supplementary material for: RLIM suppresses hepatocellular carcinogenesis by up-regulating p15 and p21
Source: Oncotarget. 2017 Sep 15;8(47):83075–87. doi: 10.18632/oncotarget.20904 (PMC5669951; doi:10.18632/oncotarget.20904)
Supplement: Supplementary file 1 [file oncotarget-08-83075-s001.pdf]

RLIM suppresses hepatocellular carcinogenesis by up-regulating p15 and p21

SUPPLEMENTARY MATERIALS

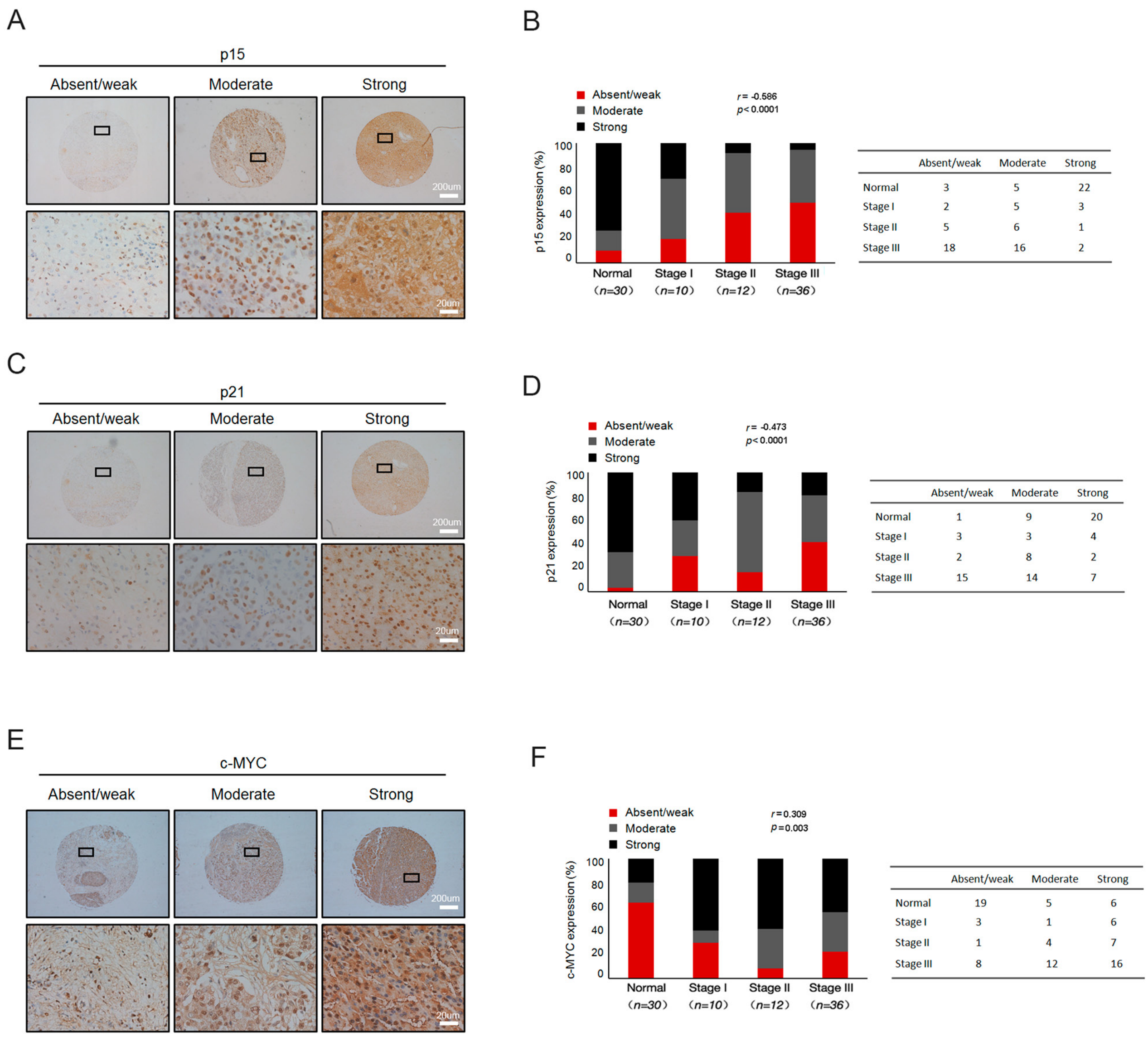

Supplementary Figure 1: The expressions of p15 and p21 negatively correlate with HCC progression, while c-MYC expressions positively correlate. (A, C, E) The representative IHC staining of p15 (A), p21 (C) and c-MYC (E) in human normal liver and HCC tissues. The expressions of RLIM were classified as absent/weak, moderate and strong. Upper images are lower magnification, and lower images are enlarged insets. Scale bars: 200 μm; 20 μm (insets). **(B, D, F)** Left panel: analysis showing the percentages of p15 (B), p21 (D) and c-MYC (F) expressions in normal liver tissues and each HCC clinical stage, with the r and p values of the Spearman rank correlation test indicated. Right panel: the number of different expressions of p15, p21 and c-MYC in human normal liver and HCC tissues.

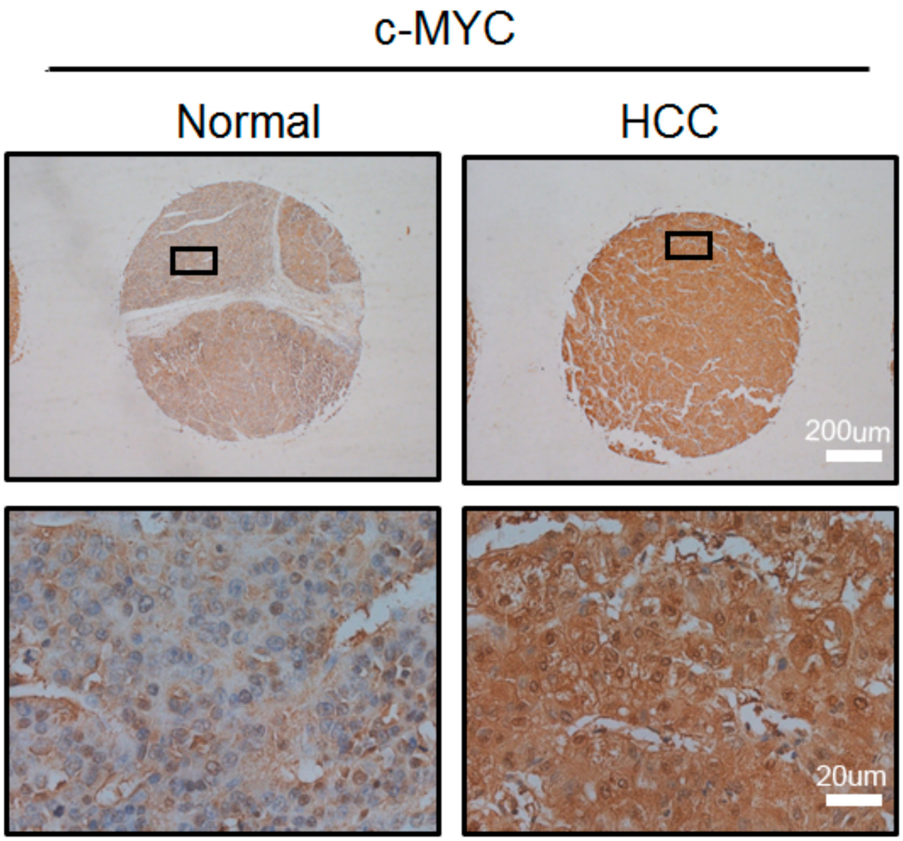

Supplementary Figure 2: c-MYC was markedly overexpressed in HCC. The representative IHC staining of c-MYC in human normal liver and HCC tissues. Upper images are lower magnification, and lower images are enlarged insets. Scale bars: 200 µm; 20 µm (insets).
